# DR. BHABESH CHANDRA LAHIRI (11.8.1932 to 5.8.2006)

**Published:** 2008

**Authors:** Arijit Coondoo

**Affiliations:** *Professor, Department of Dermatology, Vivekananda Institute of Medical Sciences, Kolkata, India. E-mail: acoondoo@gmail.com*

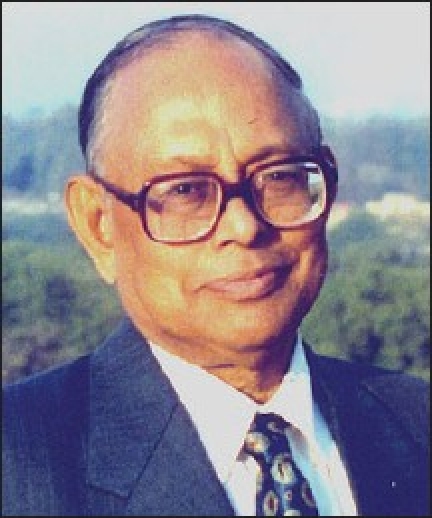



Dr. Bhabesh Chandra Lahiri was born on 11.8.1932 at Natore (now in Bangladesh) in an affluent zamindar family. His father was a renowned physician. A brilliant student, he passed his Matriculation (School Leaving Examination) at the tender age of 14 years. Subsequently he passed the I.Sc examinations from Rajshahi College and B.Sc. from City College, Calcutta before obtaining his MBBS degree from NRS Medical College, Calcutta in 1957. He took his training at the School of Tropical Medicine in 1959 for a special fellowship in Leprosy. Subsequently, he passed the D. Dermat examination of the Calcutta University in 1967.

He joined the West Bengal Health Services in 1962 and after passing the D. Dermat examinations he was posted at Bankura Sammilani Medical College where he was instrumental in starting the Department of Dermatology in 1969. He was later transferred to Burdwan Medical College in 1973. Here again he was the first teacher in the Department of Dermatology of Burdwan Medical College and was intensely associated with all the academic and teaching activities in the department till he retired in 1990. His lucid teaching, polite behavior and inherent humbleness attracted many brilliant students to the subject of dermatology. His administrative acumen was also evident when as a senior member of the faculty he served as Deputy Superintendent and Acting Superintendent in the same hospital with effortless ease. His firmness and practical approach helped him tide over many a crisis during this period.

From 1973 onwards he developed a fabulous practice in Burdwan and soon his name became synonymous with dermatology not only in Burdwan but also in all the adjoining districts of South Bengal. A founder member of IADVL, his devotion to the Association was evident from the fact that he attended almost every national, state and zonal conferences of IADVL. Due to his introvert nature he was always averse to pushing himself into the limelight. However, he had a keen intellect and an intense interest in the affairs of IADVL and was always ready at hand to advise his son Koushik when the latter was elected as the Honorary General Secretary of IADVL. Unfortunately he could not see his son complete his term. He passed away on the afternoon of August 5, 2006 after suffering a silent myocardial infarction earlier in the day. Besides Koushik, he is survived by his wife Suchhanda, a renowned Rabindrasangeet singer and daughter Sudeshna, a doctor and hospital administrator. May his soul rest in peace.

